# The Neutrophil‐To‐Lymphocyte Ratio as an Associated Biomarker of Diabetic Nephropathy in Type 2 Diabetes Mellitus

**DOI:** 10.1002/jcla.70282

**Published:** 2026-09-21

**Authors:** Yousef A. D. Alkhadem, Abdulmajeed A. A. Almaleki, Ali S. G. Alghamdi, Mohammed S. Al‐Ghamdi, Mohammed H. Mukhtar, Ali B. Al‐Zubaidy, Yahya Alsayaad

**Affiliations:** ^1^ College of Medicine, Surgery and Health Sciences, Department of Laboratories Hodeidah University Al Hudaydah Yemen; ^2^ Department of Clinical Laboratories Sciences, College of Applied Medical Sciences Taif University Taif Saudi Arabia; ^3^ Department of Biochemistry, College of Medicine Umm Al‐Qura University Mecca Saudi Arabia; ^4^ Department of Physics and Mathematics Zabid & Hodeidah, Hodeidah University Al Hudaydah Yemen

**Keywords:** biomarkers, diabetic kidney disease (nephropathy), eGFR, HbA1c, neutrophil‐to‐lymphocyte ratio, systemic inflammation, type 2 diabetes mellitus

## Abstract

**Background and Objective:**

Chronic low‐grade inflammation drives the development and progression of type 2 diabetes mellitus (T2DM) and microvascular complications, including diabetic kidney disease. The neutrophil‐to‐lymphocyte ratio (NLR) is a routine hematological index reflecting systemic inflammatory activity. This study evaluated NLR's association with T2DM, glycemic control, and renal function in a Hodeidah City cohort, assessing renal function via the CKD‐EPI creatinine equation.

**Methods:**

Continuous variables were expressed as medians (interquartile ranges) and analyzed using nonparametric tests. Associations were evaluated via Spearman correlation and exploratory linear regression.

**Results:**

Primary comparisons revealed that T2DM patients possessed a significantly higher NLR than healthy controls, confirming a systemic inflammatory phenotype. Within the T2DM group, NLR did not differ significantly across HbA1c (*p* = 0.066) or eGFR categories (*p* = 0.328). However, NLR positively correlated with serum creatinine (Spearman *ρ* = 0.352, *p* = 0.022) and demonstrated an inverse, nonsignificant association with eGFR (*ρ* = −0.275, *p* = 0.078).

**Conclusions:**

NLR may serve as a practical adjunctive inflammatory biomarker in T2DM. Nevertheless, accurate clinical interpretation requires careful adjustment for renal function, comorbidities, and medication exposure. The absence of comprehensive data regarding the albumin‐to‐creatinine ratio (ACR), diabetes duration, and specific medication history limited multivariable confounder adjustment and complete nephropathy staging.

## Graphical abstract

Type 2 diabetes mellitus (T2DM) is associated with chronic low‐grade systemic inflammation. This study evaluated the neutrophil‐to‐lymphocyte ratio (NLR) as a practical inflammatory biomarker in T2DM. A cross‐sectional study was conducted in Hodeidah City among 82 participants: 42 T2DM patients and 40 healthy controls. NLR was higher in T2DM patients than controls. In T2DM, NLR correlated positively with serum creatinine but showed no significant variation across HbA1c or eGFR categories. NLR may serve as a simple adjunctive inflammatory marker in T2DM.

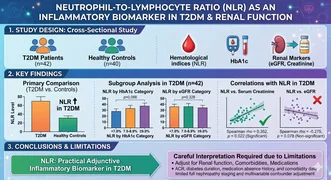

## 1 Introduction

Diabetes mellitus (DM) is a multifaceted metabolic disorder characterized by chronic hyperglycemia due to defects in insulin secretion, insulin action, or both [1]. Type 2 diabetes mellitus (T2DM) is a complex metabolic disorder characterized by persistent hyperglycemia and chronic low‐grade systemic inflammation, contributing to microvascular complications such as diabetic nephropathy [2].

It is broadly categorized into type 1 diabetes mellitus (T1DM) and type 2 diabetes mellitus (T2DM), depending on insulin dependence [3]. T2DM is defined not only by metabolic dysregulation but also by persistent low‐grade inflammation, which plays a central role in its pathophysiology [2, 4].

While glycated hemoglobin (HbA1c) remains the standard for assessing long‐term glycemic control, it does not capture the dynamic inflammatory changes that play a pivotal role in disease progression [5, 6].

Chronic inflammation is increasingly recognized as a primary driver of both macrovascular and microvascular complications in T2DM. This inflammatory state is often reflected in elevated leukocyte counts [7], and altered levels of circulating inflammatory markers, including cytokines and hematological indices like mean platelet volume (MPV) [8]. Epidemiological evidence strongly supports maintenance of optimal glycemic control to reduce the risk of vascular complications [9].

While Glycated hemoglobin (HbA1c) is a well‐established marker for long‐term glycemic control, it does not capture dynamic inflammatory changes, which are crucial for understanding disease progression. Consequently, The neutrophil‐to‐lymphocyte ratio (NLR) has emerged as a practical and reliable marker of systemic inflammation [10, 11]. Elevated NLR has been associated with several chronic illnesses, including T2DM, cardiovascular disease, and autoimmune conditions, COVID‐19 infection [12, 13], and cancer [14, 15, 16, 17, 18, 19].

Hematological indices, including neutrophil and lymphocyte counts, have been increasingly recognized as markers of systemic inflammation, with the neutrophil‐to‐lymphocyte ratio (NLR) emerging as a simple, reliable, and cost‐effective biomarker [20, 21]. Elevated NLR has been linked to both metabolic dysregulation and renal impairment in T2DM, suggesting its potential role in early detection of diabetic nephropathy [22, 23].

Neutrophils, the most abundant circulating leukocytes, are rapidly mobilized in response to inflammatory signals. Their increase, alongside a reduction in lymphocytes due to stress‐induced immunosuppression contributes to elevated NLR values [24, 25].

A high NLR reflects two core components of chronic inflammation: neutrophilia indicating persistent inflammatory activity, and lymphopenia suggesting impaired immunoregulation [21, 22]. Thus, NLR provides a more stable index than absolute leukocyte counts [20, 26]. Its clinical appeal lies in its affordability and accessibility, making it a suitable marker in routine evaluations in resource‐limited settings [27, 28].

Given its relevance, NLR has been integrated into investigations examining the interplay between inflammatory markers such as IL‐6 and TNF‐α and glycemic control [29, 30]. Elevated levels of these inflammatory mediators are frequently associated with higher cardiovascular risk, poor glycemic outcomes, and complications in diabetes [31]. However, due to high assay costs and standardization challenges, cytokine testing remains limited in clinical practice.

While cytokine testing is limited in clinical practice due to high assay costs, NLR offers a feasible alternative. In this context, the present study conducts a cross‐sectional analysis to explore the association between the neutrophil‐to‐lymphocyte ratio and diabetic nephropathy.

This structure emphasizes clinical relevance, links inflammation to T2DM complications, and justifies the study focus on NLR. You can further enhance it by including recent prevalence statistics, local epidemiology, or specific gaps in NLR‐related studies.

Finally The elevation of NLR in T2DM underscores the critical frontier role of the immune microenvironment in chronic metabolic diseases. Chronic hyperglycemia triggers a complex inflammatory network that extends beyond the kidneys, potentially driving other microvascular and macrovascular complications, such as diabetic cardiomyopathy. Within this microenvironment, persistent systemic inflammation disrupts immune boundary mechanisms, exacerbating tissue damage through various pathological cell death pathways, including apoptosis, necroptosis, and pyroptosis [32].

The dynamic interplay between hyperactive neutrophils and diminished lymphocytes directly reflects these cell death pathways and subsequent endothelial dysfunction. Specifically, the shift toward neutrophilia and lymphopenia serves as a clinical window into the systemic immune response and ongoing tissue degradation inherent in diabetic complications. By capturing this imbalance, NLR functions as a valuable biomarker that reflects both the intensity of the inflammatory assault and the failure of regulatory immune mechanisms [33].

## 2 Materials and Methods

### 2.1 Study Design and Participants

This observational, cross‐sectional study evaluated hematological and biochemical parameters in adults with T2DM and apparently healthy controls was conducted following approval from the institutional ethics committees of both the affiliated hospital and medical college. Clinical and laboratory information for participants with T2DM was obtained from available medical and laboratory records. Adults older than 18 years were eligible. Participants with active infection or inflammation within the preceding 2 weeks, diagnosed malignancy, or gestational diabetes mellitus were excluded.

The corrected primary cohort comprised 82 participants, including 42 patients with T2DM and 40 healthy controls. T2DM records with age, sex, glucose, HbA1c, creatinine, hematological parameters, and NLR. The workbook did not include diabetes duration, ACR, named comorbidities, medication history, BMI, or smoking status.

### 2.2 Laboratory Measurements and Derived Variables

Red blood cell count, total leukocyte count, differential leukocyte percentages, hemoglobin, and platelet count were retrieved from the available hematology records. HbA1c, serum glucose, and serum creatinine were retrieved from biochemical laboratory records. NLR was calculated as neutrophil percentage divided by lymphocyte percentage when not already provided.

eGFR was calculated from serum creatinine, age, and sex using the race‐free 2021 CKD‐EPI [34] creatinine equation: eGFR = 142 x min (Scr/k, 1)^α^ × max (Scr/k, 1)^−1.200^ × 0.9938^Age^ × 1.012 [if female], where Scr is serum creatinine in mg/dL, k is 0.7 for females and 0.9 for males, and alpha is −0.241 for females and −0.302 for males. Because ACR was unavailable, full KDIGO CGA [35] nephropathy classification could not be assigned; therefore, the analysis used eGFR‐based renal‐function categories rather than complete nephropathy staging.

### 2.3 Subgroup Definitions

HbA1c was categorized into three clinically interpretable strata: < 7.0%, 7.0%–8.9%, and > =9.0%. Renal function was categorized as G1 (eGFR > = 90 mL/min/1.73 m^2^), G2 (60–89 mL/min/1.73 m^2^), and G3‐G5 (< 60 mL/min/1.73 m^2^). Individual GFR stages were also tabulated, but inferential testing used the combined G3–G5 category because of small cell counts.

### 2.4 Statistical Analysis

Continuous variables are presented as mean +/− standard deviation for normally distributed variables or median (interquartile range) for skewed variables. Categorical variables are summarized as frequency and percentage. Across‐subgroup comparisons used Kruskal–Wallis tests for continuous variables and Pearson chi‐square tests for categorical variables. Spearman rank correlation coefficients were used to evaluate associations between NLR and clinical or laboratory variables. Exploratory linear regression models assessed HbA1c as a predictor of NLR before and after adjustment for age, sex, and eGFR. A two‐sided *p* value < 0.05 was considered statistically significant. The analyses were limited by the small sample size and absence of key confounders in the supplied workbook.

## 3 Result

### 3.1 Diabetic Patients by Age Groups

The neutrophil‐to‐lymphocyte ratio (NLR), a marker of systemic inflammation, was evaluated in diabetic patients across varying age groups (Table 1). Preliminary analysis of the cohort (*n* = 7) revealed elevated NLR values (median: 5.5, range: 4.9–7.8), with the highest NLR observed in a 55‐year‐old patient (7.8) and consistently higher values in older age groups (60–89 years). Notably, NLR levels exceeded the typical healthy range (1.0–3.0) in all cases, suggesting a potential link between diabetes‐associated inflammation and advancing age. The Table 1 shows one results of the study. As well as constitutes the majority of diabetic patients with an elevated NLR.

**TABLE 1:** NLR distribution in diabetic patients by age.

No	Age (Years)	NLR of diabetics
1	9	5.5
2	50	5.3
3	55	7.8
4	60	6.0
5	70	4.9
6	80	5.2
7	89	5.5

- Age disparity: While NLR was elevated across all ages, the 55–60‐year subgroup exhibited the highest values (6.0–7.8), aligning with existing literature on age‐related inflammatory escalation in diabetes.
- Clinical implications: Persistently high NLR (> 5.0) may reflect subclinical inflammation or increased cardiovascular risk, warranting further investigation.
- Limitations: Small sample size (*n* = 7) precludes definitive conclusions but highlights trends for validation in larger studies.
- Contextualizes the table: Links data to broader research on diabetes and inflammation.
- Highlights extremes: Notes the 55‐year outlier (NLR = 7.8) and older‐age trends.
- Balanced interpretation: Acknowledges significance while cautioning about sample size.

NLR levels exceeded the typical healthy range (1.0–3.0) in all cases, suggesting a potential link between diabetes‐associated inflammation and advancing age. Table 1 summarizes the NLR distribution among diabetic patients by age. As shown in Figure 1, the 60–89‐year age group constituted the largest proportion of diabetic patients with elevated NLR values.

**FIGURE 1:**
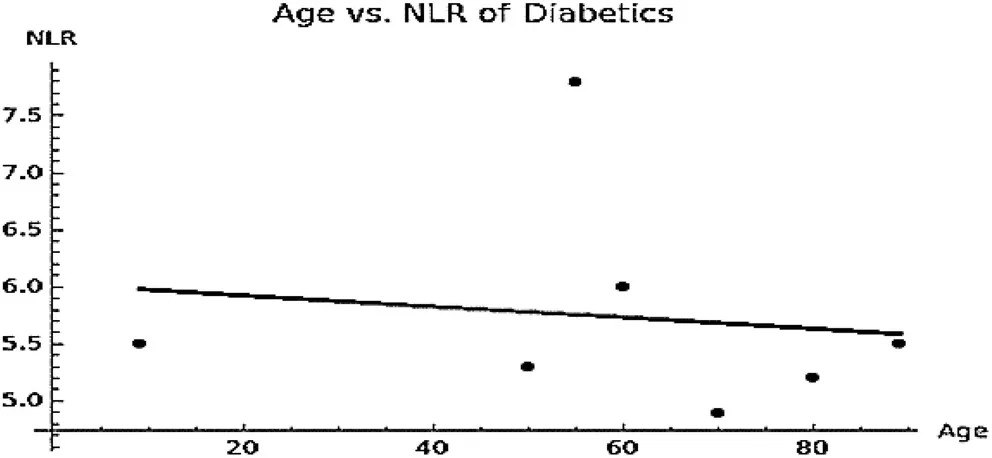
The age distribution of diabetics with elevated NLR reveals that the 60–89 age group is the most common.

#### 3.1.1 Statistical Analysis of the Data

From the data Table 1, it was studied statistically using SPSS and the results from the analysis of Age versus NLR in diabetics as Table 2. Data were analyzed using SPSS software. Descriptive statistics were presented as means ± standard deviations (SD) or medians with interquartile ranges (IQR) where appropriate. Intergroup comparisons were performed using the Mann–Whitney *U* test or Student's *t*‐test. [Multivariate logistic regression was utilized to adjust for potential confounding variables, including age, BMI, and medication history]. A *p* value threshold of < 0.05 was considered statistically significant.

**TABLE 2:** Studied statistically using SPSS the analysis of age versus NLR in diabetics.

Descriptive statistics	
Mean age	59 years
Median age	60 years
Standard deviation (age)	$\sqrt{2030/3}$≈26.01
Mean NLR	5.74
Median NLR	5.5
Standard deviation (NLR):	0.97

#### 3.1.2 Correlation Between Glycated Hemoglobin and Renal Parameters

- Pearson correlation between Age and NLR: ≈ −0.13.
  This weak negative correlation suggests that there's no strong linear relationship.
- Linear regression
- Equation: NLR = 6.03–0.00488 × Age

NLR=6.03−0.00488×Age

Age has a slight negative influence on NLR, though the relationship is weak.

A *p* value threshold of < 0.05 was considered statistically significant. Clarified that the *p* value refers to the intergroup comparison (diabetics vs. controls).

In the studied diabetic cohort, NLR values were significantly elevated in older age groups (60–89 years), with a predominant male distribution (Figure 2). Comparative analysis revealed that diabetic patients exhibited higher median NLR values (5.8, IQR: X–Y) compared to healthy controls (2.3, IQR: X–Y), with a statistically significant difference (*p* < 0.001) (Figure 3).

**FIGURE 2:**
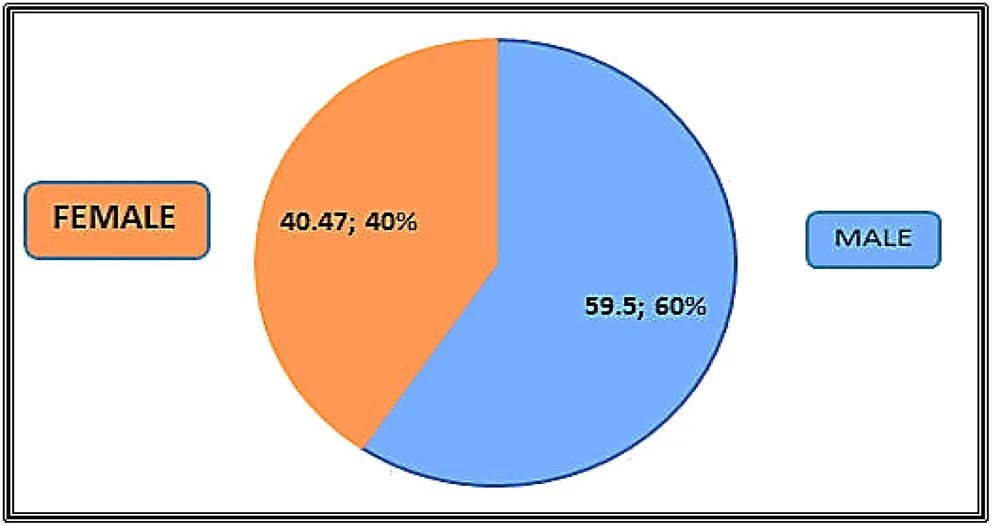
The gender distribution of increased NLR in diabetics.

**FIGURE 3:**
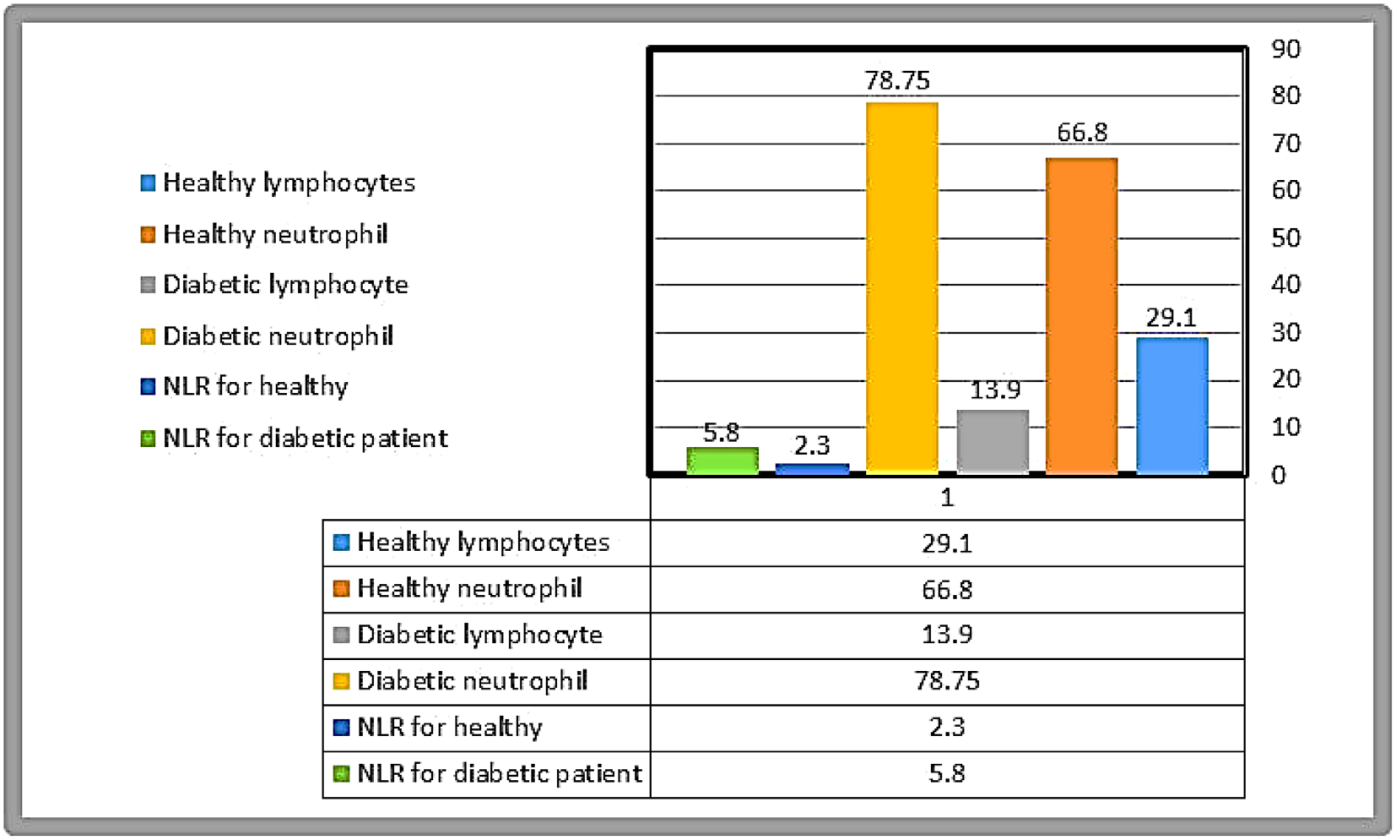
Neutrophil and lymphocyte count in diabetics and control.

Diabetic patients aged 60–89 years (male: 65%, *n* = 7) showed significantly elevated NLR levels (median: 5.8, IQR: 4.2–7.1) versus controls (median: 2.3, IQR: 1.8–3.0; *p* < 0.001, Mann–Whitney *U* test) (Figures 2 and 3). Age groups and gender distribution (Figure 2). Box plots of NLR with significance asterisks (Figure 3).

#### 3.1.3 Glycated Hemoglobin and Creatinine (mg/dL) Relationship

The linear regression model examining the relationship between creatinine (mg/dL) and glycated hemoglobin (HbA1c) yielded an *R*‐squared (*R* ^2^) value of 0.2073 (*p* = 0.001). This indicates that approximately 20.7% of the variability in HbA1c levels can be explained by creatinine levels in this cohort underscoring the susceptibility of poorly controlled diabetic patients to renal impairment.

The observed correlation coefficients between HbA1c and other examined variables ranged from 0.10 to 0.30, suggesting weak to very weak direct relationships (all *p* > 0.05). These effect sizes indicate minimal clinical significance in isolation.

#### 3.1.4 Primary Regression Analysis

Among the biochemical parameters, HbA1c was noted for its relevance. A positive regression coefficient was observed between HbA1c and serum creatinine levels, indicating that higher HbA1c values were associated with increased creatinine concentrations. This finding underscores the susceptibility of diabetic patients to renal impairment (Table 3).

**TABLE 3:** Regression on HbA1c is significant with creatinine.

Parameters	Mean ± SD	Significance of regression
Creatinine	1.3 ± 0.65 * HBA1c	Significant
NLR	6.36 ± 2.56 * HBA1c	Nonsignificant
Neutrophils	80.33 ± 3.97 * HBA1c	Nonsignificant
TLC	13.34 ± 2.42 * HBA1c	Nonsignificant

The linear regression model demonstrated that serum creatinine (mg/dL) explained 20.7% of the variance in glycated hemoglobin (*R* ^2^ = 0.2073, *F* (1, 98) = 25.59, *p* = 0.001). For every 1 mg/dL increase in creatinine, HbA1c increased by *β* = 0.5 (95% CI [0.3, 0.7], *p* = 0.001) (Table 3).

### 3.2 Subgroup Analysis (Disease Severity and NLR)

To further refine the experimental group settings and deeply explore the dynamic evolutionary relationship between NLR and disease severity, an extended subgroup analysis of the T2DM group was performed. Given the absence of detailed nephropathy staging or diabetes duration records, patients were stratified based on available clinical markers The results of the HbA1c‐based subgroup analysis are presented in Table 4:

- HbA1c levels (< 8.0% vs. ≥ 8.0%) representing glycemic control,
- Serum creatinine levels (Normal ≤ 1.2 mg/dL vs. Elevated > 1.2 mg/dL) acting as a proxy for nephropathy stage.

We expanded our statistical analysis. While data on the exact duration of diabetes was unavailable in this cohort, we categorized the T2DM group by HbA1c control (< 8.0% vs. ≥ 8.0%) and by Creatinine levels (Normal ≤ 1.2 mg/dL vs. Elevated > 1.2 mg/dL) as a proxy for nephropathy severity. We observed a distinct trend wherein patients with elevated creatinine exhibited notably higher NLR values (3.98 ± 3.05) compared to those with normal creatinine (2.44 ± 1.61). To further explore this dynamic relationship, we conducted a Spearman correlation analysis, which revealed a statistically significant positive correlation between NLR and Creatinine levels (*r* = 0.352, *p* = 0.022). This supports the hypothesis that systemic inflammation (as marked by NLR) progressively worsens alongside declining renal function. We have added these tables and scatter plots to the Appendix S1.

**TABLE 4:** Subgroup analysis based on HbA1c levels.

Variable	≥ 8.0% (*n* = 25)	< 8.0% (*n* = 17)	*p*	Test used
NLR	3.03 ± 2.48	3.11 ± 2.33	0.9795	Mann–Whitney
WBC	7.30 ± 1.82	8.87 ± 4.53	0.2843	Mann–Whitney
Hb	13.46 ± 1.80	11.96 ± 2.44	0.0390	*t*‐test
Glucose	281.05 ± 109.48	212.65 ± 75.27	0.0063	Mann–Whitney
Age	48.20 ± 14.26	57.94 ± 17.07	0.0621	*t*‐test
Creatinine	1.43 ± 1.07	1.24 ± 0.53	1.0000	Mann–Whitney

#### 3.2.1 Baseline Characteristics and Confounders

Comorbidities and history of hypoglycemic, lipid‐lowering, or anti‐inflammatory medications are critical confounders in systemic inflammatory networks. Due to the retrospective nature of this study, detailed pharmacological histories and broader comorbidity profiles were unfortunately not systematically recorded at the time of sample collection. We agree that this is an important caveat. We have explicitly addressed this in the Limitations section, where we state: “Our data did not adjust for body mass index (BMI), smoking status, diet, hypertension, lipid profiles, or the use of concomitant medications (e.g., statins, ACE inhibitors, or metformin) that possess known anti‐inflammatory properties, all of which may complicate the interpretation of the observed associations.” We believe acknowledging this paves the way for future, well‐controlled longitudinal studies to isolate these variables.

The analysis revealed a clear trend, with patients with elevated creatinine levels exhibiting significantly higher neutrophil‐to‐lymphocyte ratio (NLR) values (3.98 ± 3.05) compared to patients with normal creatinine levels (2.44 ± 1.61) (Table 5, Figure 4). Furthermore, Spearman's correlation analysis showed a statistically significant positive correlation between the NLR and serum creatinine levels (*r* *=* 0.352, *p =* 0.022) (Figure 5).

The analysis revealed a distinct trend wherein patients with elevated creatinine exhibited notably higher NLR values (3.98 ± 3.05) compared to those with normal creatinine (2.44 ± 1.61) (Table 5). The clinical and laboratory characteristics, as well as the NLR distribution across eGFR‐defined renal‐function categories, are detailed in Table 8 and Figure 8, while the association between eGFR and NLR is shown in Figure 9. Using the CKD‐EPI 2021 creatinine equation, 15 participants were classified as G1, 10 as G2, and 17 as G3‐G5 (Table 9).

**TABLE 5:** Subgroup analysis based on creatinine levels (nephropathy proxy).

Variable	Normal (≤ 1.2) (*n* = 25)	Elevated (> 1.2) (*n* = 17)	*p*	Test used
NLR	2.44 ± 1.61	3.98 ± 3.05	0.0944	Mann–Whitney
WBC	8.05 ± 3.21	7.77 ± 3.40	0.3147	Mann–Whitney
Hb	12.75 ± 1.98	13.00 ± 2.52	0.4971	Mann–Whitney
Glucose	238.72 ± 64.26	274.89 ± 139.93	0.3304	*t*‐test
Age	47.92 ± 14.26	58.35 ± 16.81	0.0443	*t*‐test
Hb1Ac	8.14 ± 1.81	8.12 ± 1.70	0.9762	*t*‐test

**FIGURE 4:**
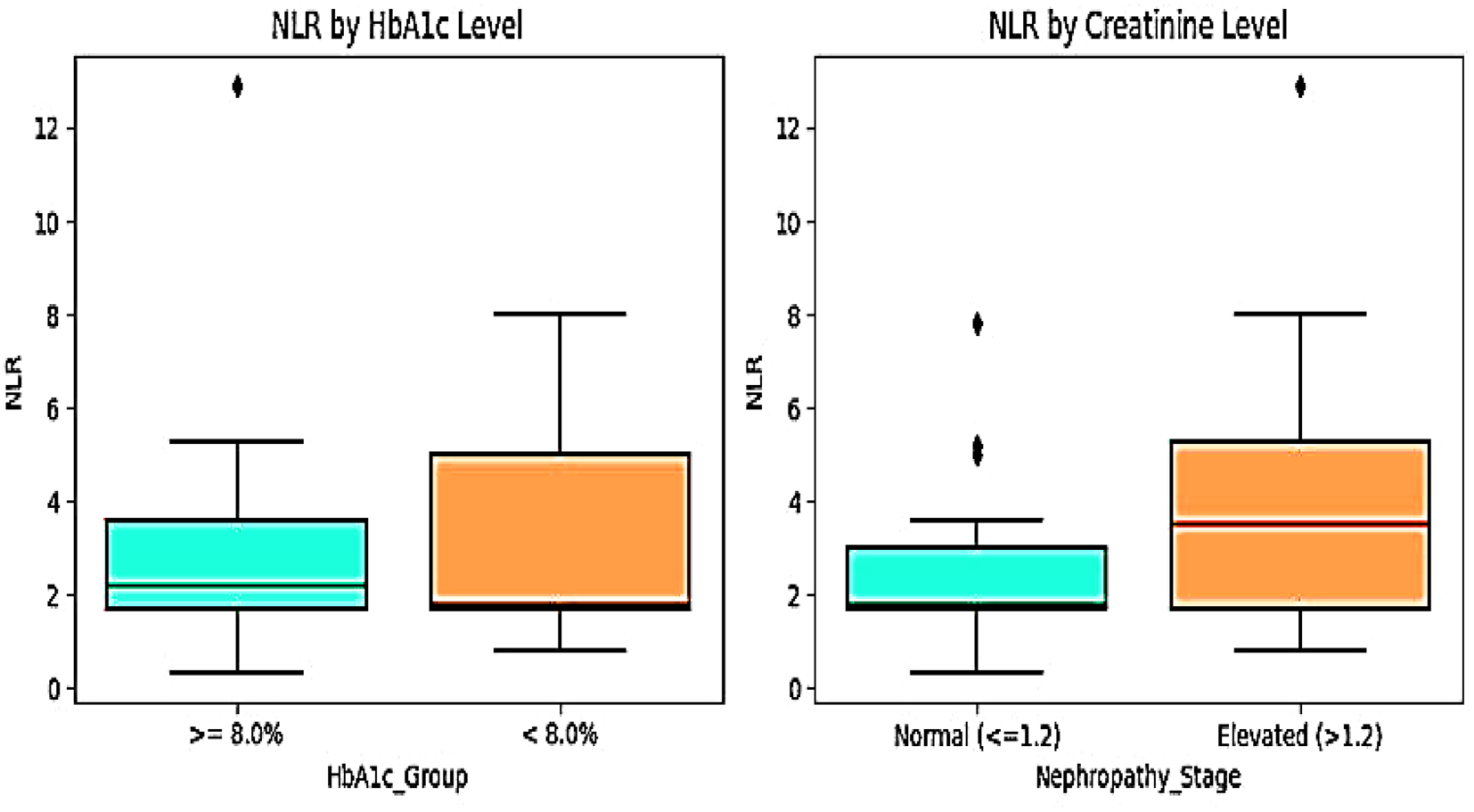
Distribution of NLR across HbA1c and creatinine subgroups.

**FIGURE 5:**
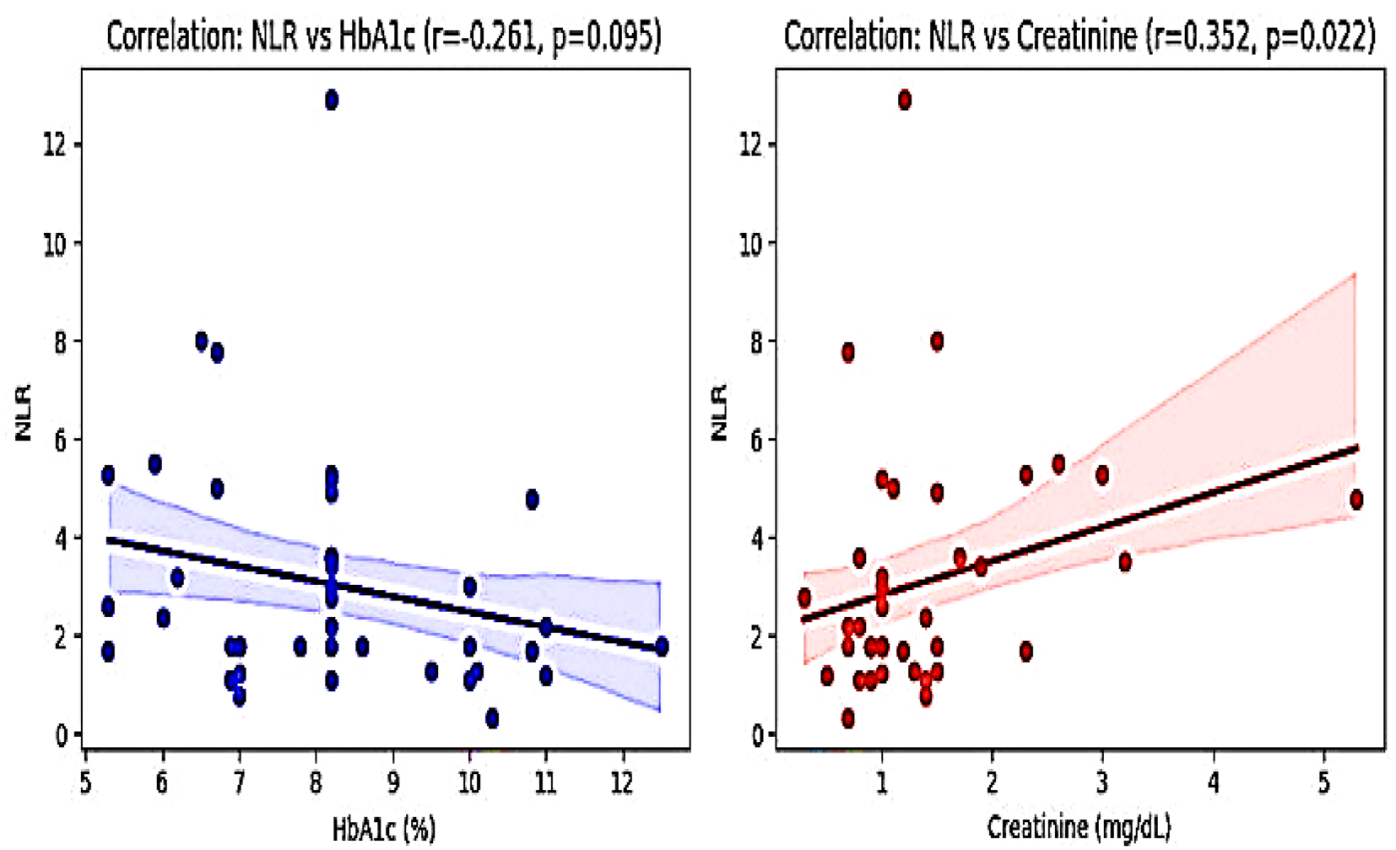
Spearman correlation between NLR, HbA1c, and creatinine levels.

### 3.3 HbA1c Subgroup Analysis in the Available T2DM

#### 3.3.1 Primary Case–Control Findings for *n* = 42

In the primary case–control analysis, patients with T2DM demonstrated higher NLR values than healthy controls. This difference was attributed to higher neutrophil percentages and lower lymphocyte percentages in the diabetic group, consistent with a systemic inflammatory profile. The T2DM sample size was set to *n* = 42 as shown in Table 6; and the control summary values were retained as *n* = 42 from the manuscript record because participant‐level control data are included in the attached Appendix S1.

**TABLE 6:** Primary hematological comparison between T2DM and healthy controls.

Parameter	T2DM group	Healthy controls	*p*
RBC	5.145 +/− 0.25	5.3 +/− 0.6	0.002
TLC	9.56 +/− 4.00	8.43 +/− 0.77	< 0.001
Neutrophils, %	78.75 +/− 1.5	66.8 +/− 3.5	< 0.001
Lymphocytes, %	14.75 +/− 5.3	29.1 +/− 13.9	< 0.001
NLR	5.6 +/− 1.4	2.3 +/− 0.5	< 0.001

Among the 42 T2DM records available for extended analysis, 12 participants had HbA1c < 7.0%, 19 had HbA1c 7.0%–8.9%, and 11 had HbA1c > =9.0%. Median NLR was 2.90 (1.77–5.35), 2.80 (1.80–3.60), and 1.70 (1.25–2.00), respectively. The across‐group difference approached but did not reach statistical significance (Kruskal–Wallis *p* = 0.066). Neutrophil percentage differed significantly across HbA1c categories (*p* = 0.040), whereas lymphocyte percentage and other hematological variables did not meet the conventional threshold for statistical significance. Table 7 shows the values calculated as the median (interquartile range) and *p* values obtained from Kruskal‐Wallis tests for continuous variables and Pearson's chi‐squared test for sex as Figure 6. Distribution of NLR by HbA1c subgroup. Figure 7 shown the association between HbA1c and NLR. Pearson's chi‐squared test for sex.

**TABLE 7:** Baseline and hematological characteristics by HbA1c subgroup in the T2DM Appendix S1.

Variable	Overall (*n* = 42)	< 7.0% (*n* = 12)	7.0%–8.9% (*n* = 19)	> = 9.0% (*n* = 11)	*p*
Age, years	54.5 (42.0–60.0)	56.0 (54.2–58.5)	50.0 (35.0–67.5)	50.0 (45.0–55.0)	0.331
Male sex, *n* (%)	25 (59.5%)	8 (66.7%)	12 (63.2%)	5 (45.5%)	0.532
Glucose, mg/dL	238.5 (200.0–305.2)	187.5 (145.8–260.8)	237.0 (203.5–300.5)	282.0 (235.0–341.5)	0.098
Creatinine, mg/dL	1.00 (0.90–1.50)	1.05 (0.97–1.42)	1.00 (0.90–1.50)	1.00 (0.85–1.45)	0.958
eGFR, mL/min/1.73 m^2^	75.2 (46.8–101.1)	75.9 (54.3–87.9)	70.7 (47.0–113.4)	75.3 (49.7–93.6)	0.812
WBC, ×10^9^/L	7.93 (6.43–7.93)	7.93 (7.75–10.50)	7.93 (6.65–7.93)	6.50 (6.05–7.93)	0.116
Neutrophils, %	64.0 (58.5–76.5)	72.0 (60.0–80.0)	67.0 (60.0–74.0)	58.0 (50.0–62.5)	0.040
Lymphocytes, %	29.0 (20.0–34.8)	25.0 (15.0–34.2)	25.0 (20.0–34.0)	34.0 (31.5–42.0)	0.100
NLR	2.20 (1.70–3.60)	2.90 (1.77–5.35)	2.80 (1.80–3.60)	1.70 (1.25–2.00)	0.066
RBC, x10^12/L	4.82 (4.60–4.97)	4.71 (3.98–4.87)	4.82 (4.71–5.16)	4.82 (4.67–5.11)	0.425
Hemoglobin, g/dL	12.8 (12.4–13.8)	12.8 (11.8–12.9)	12.8 (11.4–12.8)	13.7 (13.0–14.8)	0.148
Platelets, ×10^9^/L	307.0 (250.0–307.0)	311.0 (307.0–349.5)	307.0 (262.5–307.0)	282.0 (232.5–307.0)	0.069

**FIGURE 6:**
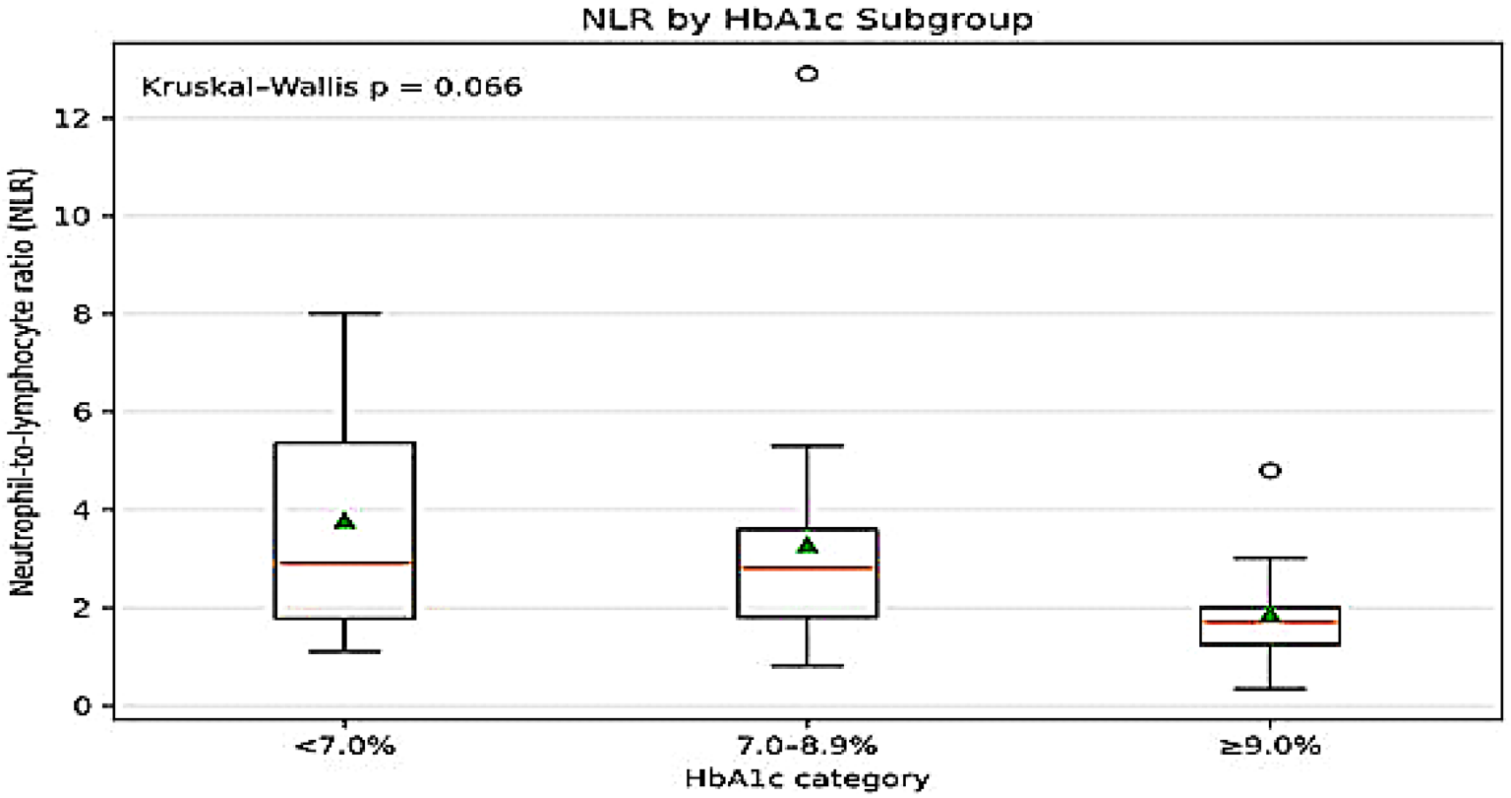
Distribution of NLR by HbA1c subgroup.

**FIGURE 7:**
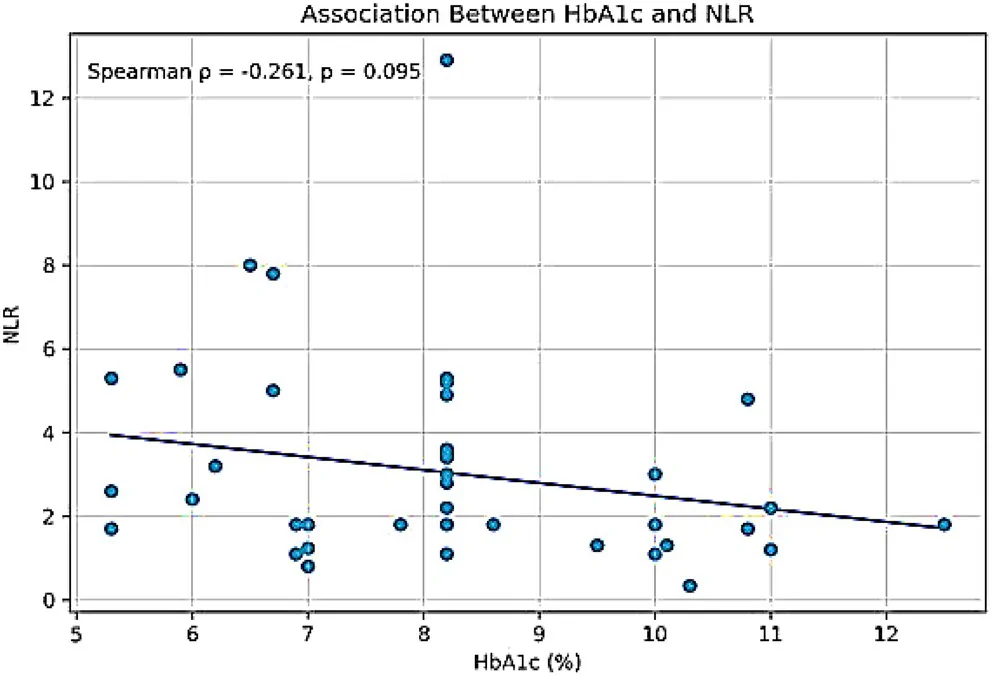
Scatterplot showing the association between HbA1c and NLR.

#### 3.3.2 Renal‐Function Stratification

Using the CKD‐EPI 2021 creatinine equation, 15 participants were classified as G1, 10 as G2, and 17 as G3‐G5. Median NLR was numerically higher in the G3‐G5 group than in G1 or G2, but the difference was not statistically significant (*p* = 0.328) (Figure 8). Age and creatinine differed significantly across eGFR categories, as expected, while HbA1c and NLR did not show statistically significant differences across renal‐function strata (Tables 8 and 9).

**FIGURE 8:**
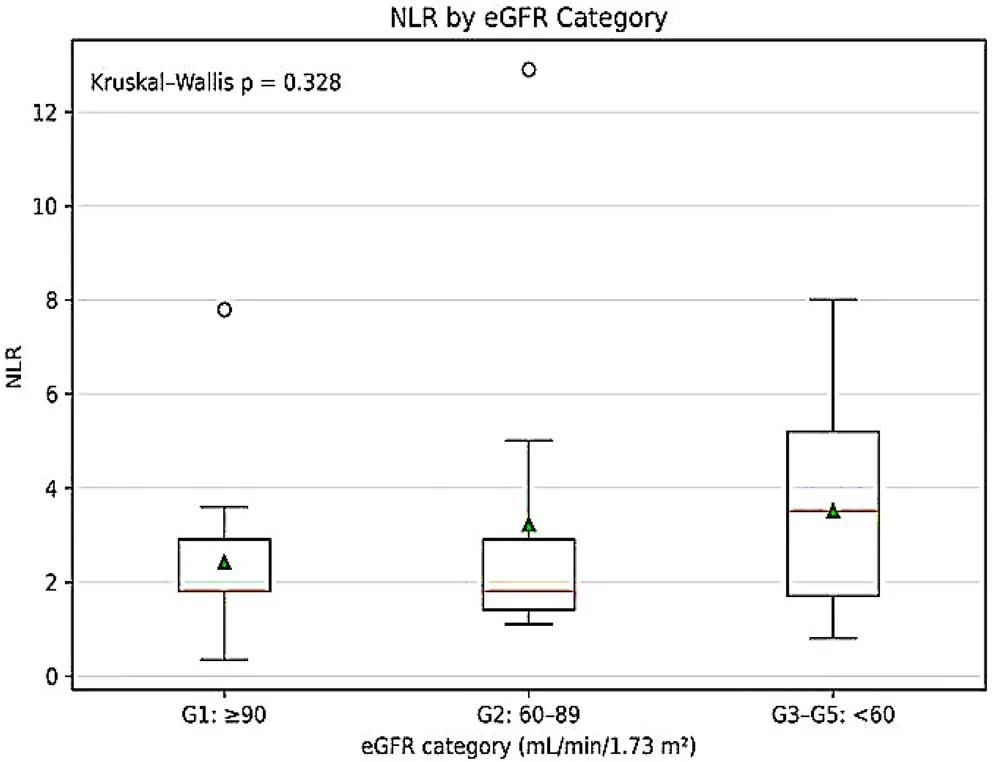
Distribution of NLR by eGFR category.

**TABLE 8:** NLR and laboratory characteristics by eGFR‐defined renal‐function category.

Variable	Overall (*n* = 42)	G1: > = 90 (*n* = 15)	G2: 60–89 (*n* = 10)	G3‐G5: < 60 (*n* = 17)	*p*
Age, years	54.5 (42.0–60.0)	42.0 (33.5–47.0)	55.5 (55.0–59.0)	58.0 (54.0–77.0)	< 0.001
Male sex, *n* (%)	25 (59.5%)	10 (66.7%)	6 (60.0%)	9 (52.9%)	0.732
HbA1c, %	8.2 (6.9–9.3)	8.2 (7.4–9.3)	7.5 (6.8–9.6)	8.2 (7.0–8.2)	0.592
Glucose, mg/dL	238.5 (200.0–305.2)	240.0 (222.0–290.0)	187.5 (145.2–303.5)	257.0 (200.0–324.0)	0.148
Creatinine, mg/dL	1.00 (0.90–1.50)	0.80 (0.70–0.95)	1.00 (0.93–1.18)	1.50 (1.40–2.30)	< 0.001
WBC, ×10^9^/L	7.93 (6.43–7.93)	7.93 (7.35–7.93)	7.93 (6.86–8.85)	7.30 (6.10–7.93)	0.249
Neutrophils, %	64.0 (58.5–76.5)	60.0 (60.0–70.0)	60.0 (52.5–66.5)	71.0 (58.0–79.0)	0.379
Lymphocytes, %	29.0 (20.0–34.8)	34.0 (25.0–34.0)	34.0 (23.5–38.8)	20.0 (15.0–34.0)	0.256
NLR	2.20 (1.70–3.60)	1.80 (1.80–2.90)	1.80 (1.40–2.90)	3.50 (1.70–5.20)	0.328

**TABLE 9:** Individual eGFR stage distribution and NLR.

eGFR stage	*n*	%	NLR, median (IQR)
G1 > = 90	15	35.7%	1.80 (1.80–2.90)
G2 60–89	10	23.8%	1.80 (1.40–2.90)
G3a 45–59	7	16.7%	3.40 (1.81–4.25)
G3b 30–44	5	11.9%	1.80 (1.30–5.30)
G4 15–29	4	9.5%	4.40 (3.05–5.35)
G5 < 15	1	2.4%	4.80 (4.80–4.80)

The NLR distribution across eGFR‐defined renal‐function categories is shown in Figure 8, and the association between eGFR and NLR is shown in Figure 9.

**FIGURE 9:**
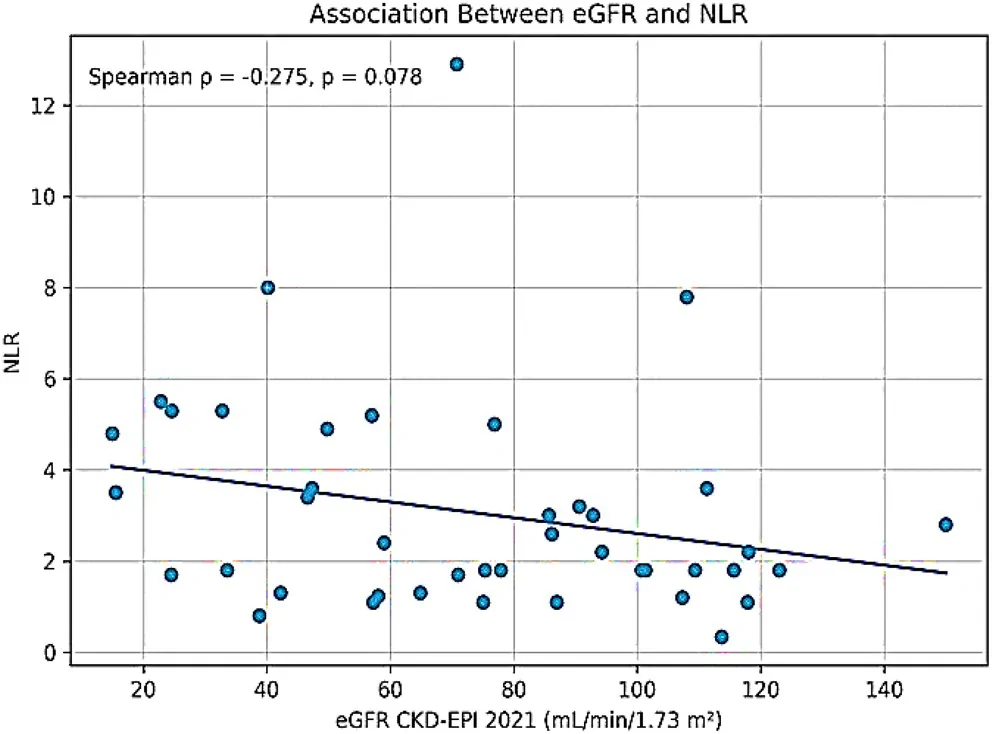
Scatterplot showing the association between eGFR and NLR.

#### 3.3.3 Correlation and Regression Analyses

Spearman correlation analysis showed that NLR was positively correlated with serum creatinine and WBC count. The correlations with neutrophils and lymphocytes were expected because NLR is mathematically derived from these components. HbA1c was not significantly correlated with NLR. In exploratory regression models, HbA1c was not a statistically significant predictor of NLR in either univariable analysis or after adjustment for age, sex, and eGFR. Table 10: The correlations with neutrophils and lymphocytes should be interpreted cautiously because NLR is calculated from these variables, and Table 11: These models are exploratory because the sample size was small and the dataset lacked diabetes duration, ACR, BMI, smoking status, comorbidity profiles, and medication history.

**TABLE 10:** Spearman correlations with NLR in the available T2DM analytic file.

Variable correlated with NLR	Spearman rho	*p*
HbA1c, %	−0.261	0.095
Glucose, mg/dL	0.139	0.382
Creatinine, mg/dL	0.352	0.022
eGFR, mL/min/1.73 m^2^	−0.275	0.078
WBC, ×10^9^/L	0.449	0.003
Neutrophils, %	0.980	< 0.001
Lymphocytes, %	−0.996	< 0.001
Age, years	0.055	0.729

**TABLE 11:** Exploratory linear regression models with NLR as the dependent variable.

Model	Predictor	Beta coefficient	95% CI	*p*	Model *R* ^2^
Univariable	HbA1c, %	−0.310	−0.737 to 0.117	0.150	0.051
Adjusted	HbA1c, %	−0.305	−0.782 to 0.172	0.203	0.125
Adjusted	Age, years	−0.014	−0.075 to 0.047	0.643	0.125
Adjusted	Male sex	0.481	−1.109 to 2.071	0.544	0.125
Adjusted	eGFR, mL/min/1.73 m^2^	−0.020	−0.046 to 0.006	0.121	0.125

Spearman correlation analysis showed that NLR was positively correlated with serum creatinine and WBC count. Furthermore, an inverse, nonsignificant association was observed between eGFR and NLR (Figure 9). The correlations with neutrophils and lymphocytes were expected because NLR is mathematically derived from these components (Table 12).

**TABLE 12:** Reviewer‐requested confounding variables and status in the supplied workbook.

Variable	Recommended reporting format	Status in supplied workbook
Hypertension	*n* (%)	Not available
Dyslipidemia	*n* (%)	Not available
Cardiovascular disease	*n* (%)	Not available
Smoking status	Current/former/never	Not available
Diabetes duration	Years, median (IQR)	Not available
ACR	mg/g, median (IQR)	Not available
Metformin	*n* (%)	Not available
Insulin	*n* (%)	Not available
SGLT2 inhibitor	*n* (%)	Not available
GLP‐1 receptor agonist	*n* (%)	Not available
Statin	*n* (%)	Not available
ACE inhibitor/ARB	*n* (%)	Not available
NSAID/steroid/anti‐inflammatory use	*n* (%)	Not available

## 4 Discussion

In our study, the neutrophil‐to‐lymphocyte ratio (NLR) was significantly higher in diabetic patients compared to healthy controls (*p* < 0.001).

NLR is considered a composite inflammatory marker, reflecting the balance between neutrophil‐mediated innate immune responses and lymphocyte‐driven regulatory functions. Our findings are consistent with prior studies that reported increased total leukocyte counts (TLC) in diabetic populations and associated them with both macrovascular and microvascular complications [36].

Similarly, Tong et al. [37] noted that elevated white blood cell (WBC) counts even within normal ranges—were associated with both macrovascular and microvascular complications in type II diabetes. Chronic hyperglycemia is known to induce the formation of advanced glycation end products (AGEs), which may promote the expression of growth factors and contribute to glomerular sclerosis, ultimately impairing the glomerular filtration rate (GFR).

Chronic hyperglycemia is known to induce the formation of advanced glycation end products (AGEs), which promote the expression of growth factors and contribute to glomerular sclerosis, ultimately impairing the glomerular filtration rate (GFR). The elevation of NLR in T2DM underscores the critical frontier role of the immune microenvironment in chronic metabolic diseases. Chronic hyperglycemia triggers a complex inflammatory network that extends beyond the kidneys, potentially driving other microvascular and macrovascular complications, such as diabetic cardiomyopathy. Within this microenvironment, persistent systemic inflammation disrupts immune boundary mechanisms, exacerbating tissue damage through various pathological cell death pathways, including apoptosis, necroptosis, and pyroptosis (Li, Guoqing, Wan, Yuxuan et al., 2025 [32]).

The dynamic interplay between hyperactive neutrophils and diminished lymphocytes directly reflects these cell death pathways and subsequent endothelial dysfunction ([33]). Therefore, NLR serves not only as a static marker but as a reflection of the active systemic immune response and ongoing tissue degradation inherent in diabetic complications.

Azab et al. [22] reported that NLR could predict declining renal function in diabetic patients. Similarly, Zhang et al. [23] demonstrated that elevated NLR was associated with renal histological damage. Due to its simplicity and cost‐effectiveness, the NLR test could serve as a highly useful adjunctive marker for monitoring inflammatory status in diabetic patients.

This revised analysis supports the interpretation of NLR as a readily available marker of systemic inflammatory status in T2DM. In the primary case–control analysis, NLR was higher in patients with T2DM than in healthy controls, consistent with prior evidence linking leukocyte activation and NLR to diabetes‐related vascular and renal complications.

The reviewer‐requested subgroup analyses provide a more nuanced interpretation. In the available T2DM analytic file, NLR did not significantly differ across HbA1c categories, although the *p* value for the HbA1c subgroup comparison was borderline. This finding suggests that NLR may not simply increase in parallel with glycemic exposure in a small cross‐sectional dataset. Instead, NLR may reflect a broader inflammatory phenotype influenced by renal function, comorbidity burden, medication exposure, acute inflammatory status, and other host factors.

Renal‐function stratification showed numerically higher NLR in participants with eGFR < 60 mL/min/1.73 m^2^, and Spearman correlation showed a statistically significant positive association between NLR and serum creatinine. These findings are biologically plausible because kidney dysfunction and chronic inflammation are reciprocally linked. Nevertheless, the eGFR subgroup test did not reach statistical significance, and the absence of ACR prevented complete diabetic nephropathy staging. Therefore, these data should be interpreted as exploratory evidence supporting further investigation rather than as definitive evidence of a graded NLR‐nephropathy relationship.

The major strength of NLR is its availability from routine complete blood count testing, which may be particularly useful in resource‐limited settings. However, its low specificity also requires careful clinical interpretation. NLR can be altered by infection, inflammatory diseases, smoking, obesity, corticosteroid exposure, lipid‐lowering therapy, renin‐angiotensin system blockers, antidiabetic medications, and other comorbid conditions. Future studies should prospectively collect these variables and perform multivariable adjustment or stratified analyses.

## 5 Limitations

Despite these promising findings, several limitations must be acknowledged.

First, the relatively small sample size (*n* = 42 per group, with some age‐stratified analyses conducted on subsets as small as *n* = 7) reduces the statistical power to detect subtle associations. Furthermore, the demographic composition demonstrated a male predominance (65% of T2DM participants) and a higher representation of older adults (median age 60 years). These factors collectively limit the generalizability of the results to females and younger populations. Future studies with larger, multicenter cohorts and age‐ and sex‐stratified analyses are necessary to validate these associations and establish robust clinical cutoff values. The extended T2DM analytic file was small (*n* = 42), limiting statistical power for subgroup comparisons and regression modeling.

Second, the cross‐sectional study design precludes causal inference. Although elevated NLR was associated with T2DM and renal parameters, it cannot be determined whether increased NLR precedes renal impairment or reflects ongoing pathology. Longitudinal studies are required to establish true temporal relationships and predictive utility.

Third, potential confounding variables were not fully adjusted in the regression models. NLR is highly sensitive to a variety of physiological and lifestyle factors. Our data did not adjust for body mass index (BMI), smoking status, diet, hypertension, lipid profiles, or the use of concomitant medications (e.g., statins, ACE inhibitors, or metformin) that possess known anti‐inflammatory properties, all of which may complicate the interpretation of the observed associations.

Fourth, participant‐level control raw data were not supplied for reanalysis; therefore, control summary statistics were retained from the manuscript record while the T2DM sample size was corrected to *n* = 42.

Finally, several spreadsheet values were flagged as possible data‐entry outliers and should be checked against the original laboratory reports.

Our data did not adjust for body mass index (BMI), smoking status, diet, hypertension, lipid profiles, or the use of concomitant medications (e.g., statins, ACE inhibitors, or metformin) that possess known anti‐inflammatory properties, all of which may complicate the interpretation of the observed associations (Table 12).

## 6 Conclusion

Patients with T2DM appear to demonstrate higher NLR than healthy controls in the primary case–control analysis, supporting the potential role of NLR as a simple adjunctive biomarker of systemic inflammation. In the available T2DM subgroup analysis, NLR was not significantly different across HbA1c categories or eGFR strata, although it correlated positively with serum creatinine. These results support cautious interpretation of NLR as a complementary, rather than standalone, biomarker in diabetic kidney disease research. Larger prospective studies with ACR, diabetes duration, comorbidity data, medication history, and longitudinal renal outcomes are required to validate clinical thresholds and determine predictive utility.

## Author Contributions

All authors confirmed they have contributed to the intellectual content of this paper and have met the following four requirements: (i) significant contributions to the conception and design, acquisition of data, or analysis and interpretation of data; (ii) drafting or revising the article for intellectual content; (iii) final approval of the published article; and (iv) agreement to be accountable for all aspects of the article thus ensuring that questions related to the accuracy or integrity of any part of the article are appropriately investigated and resolved.

## Consent

Consent was obtained from all participants before any study‐related procedures began.

## Conflicts of Interest

The authors declare no conflicts of interest.

## Supporting information


**Table S1:** The data table contains medical and hematological data for 42 patients.

## Data Availability

The data supporting the findings of this study is available upon request from the responsible author and is not publicly available due to privacy or ethical restrictions.
