# Supplementary material for: The Neutrophil‐To‐Lymphocyte Ratio as an Associated Biomarker of Diabetic Nephropathy in Type 2 Diabetes Mellitus
Source: J Clin Lab Anal. 2026 Sep 21;40(19):e70282. doi: 10.1002/jcla.70282 (PMC13592231; doi:10.1002/jcla.70282)
Supplement: Supplementary file 1 [file JCLA-40-e70282-s001.docx]

**Supplementary attached file**

| WBC | RBC | platelet | Hb | NLR | Creatinine | Hb1Ac | Glucose | Sex | Age |
| --- | --- | --- | --- | --- | --- | --- | --- | --- | --- |
| 7.93 | **4.82** | **307** | **12.85** | **5.2** | **1** | **8.2** | **306** | **F** | **80** |
| 7.93 | **4.82** | **307** | **12.85** | **4.9** | **1.5** | **8.2** | **324** | **M** | **70** |
| 7.93 | **4.82** | **307** | **12.85** | **5** | **1.1** | **6.7** | **354** | **M** | **60** |
| 7.93 | **4.82** | **307** | **12.85** | **3.6** | **0.8** | **8.2** | **237** | **M** | **45** |
| 7.93 | **4.82** | **307** | **12.85** | **1.8** | **0.9** | **8.2** | **219** | **M** | **33** |
| 7.93 | **4.82** | **307** | **12.85** | **2.8** | **0.3** | **8.2** | **207** | **F** | **26** |
| 12 | **78** | **225** | **14.8** | **4.8** | **5.3** | **10.8** | **240** | **M** | **20** |
| 7.93 | **4.82** | **307** | **12.85** | **1.1** | **0.9** | **6.9** | **146** | **F** | **56** |
| 6.9 | **5.8** | **180** | **17.5** | **1.1** | **0.9** | **8.2** | **303** | **M** | **30** |
| 6.4 | **5.32** | **277** | **14.7** | **0.34** | **0.7** | **10.3** | **230** | **M** | **48** |
| 6.4 | **4.07** | **197** | **12** | **0.8** | **1.4** | **7** | **200** | **F** | **77** |
| 7.8 | **4.6** | **395** | **9.4** | **3.2** | **1** | **6.2** | **308** | **M** | **52** |
| 8.9 | **4.9** | **793** | **13.9** | **1.8** | **0.9** | **10** | **195** | **F** | **50** |
| 3.4 | **2.6** | **287** | **7.2** | **1.8** | **1.5** | **7** | **210** | **F** | **87** |
| 9.4 | **3.9** | **514** | **9** | **1.8** | **0.7** | **7.8** | **225** | **F** | **25** |
| 7.5 | **4.75** | **498** | **13.2** | **1.7** | **2.3** | **10.8** | **300** | **F** | **55** |
| 7.93 | **4.82** | **307** | **12.85** | **1.8** | **1** | **6.9** | **245** | **M** | **34** |
| 6 | **4.3** | **192** | **13.4** | **1.2** | **0.5** | **11** | **345** | **F** | **60** |
| 7.93 | **4.82** | **307** | **12.85** | **2.2** | **0.8** | **11** | **282** | **F** | **42** |
| 2.8 | **4.49** | **230** | **10.9** | **1.23** | **1** | **7** | **200** | **F** | **77** |
| 7.93 | **4.82** | **307** | **12.85** | **1.8** | **1** | **12.5** | **338** | **F** | **35** |
| 7.6 | **5.28** | **241** | **13.1** | **8** | **1.5** | **6.5** | **222** | **F** | **58** |
| 6.1 | **5.5** | **240** | **15.5** | **1.1** | **1.4** | **10** | **200** | **M** | **61** |
| 3.7 | **4.1** | **8.2** | **9** | **3** | **1** | **10** | **254** | **M** | **48** |
| 18.7 | **4.6** | **378** | **12.3** | **7.8** | **0.7** | **6.7** | **195** | **F** | **46** |
| 7.93 | **4.82** | **307** | **12.85** | **3.5** | **3.2** | **8.2** | **395** | **F** | **65** |
| 2.8 | **5.8** | **192** | **14.9** | **3.6** | **1.7** | **8.2** | **257** | **M** | **54** |
| 7.3 | **7.2** | **285** | **15.8** | **3.4** | **1.9** | **8.2** | **298** | **M** | **35** |
| 6.1 | **5.5** | **240** | **15.5** | **3** | **1** | **8.2** | **200** | **M** | **61** |
| 7.93 | **4.82** | **307** | **12.85** | **2.2** | **0.7** | **8.2** | **240** | **M** | **42** |
| 18 | **4** | **315** | **12.7** | **5.3** | **2.3** | **5.3** | **118** | **M** | **55** |
| 8.7 | **5** | **307** | **12.9** | **1.7** | **1.2** | **5.3** | **118** | **M** | **56** |
| 6.5 | **4.9** | **282** | **14.9** | **1.3** | **1.3** | **10.1** | **346** | **M** | **55** |
| 5.7 | **4.6** | **284** | **13.7** | **1.3** | **1.5** | **9.5** | **360** | **F** | **50** |
| 7.2 | **6.2** | **201** | **17** | **2.4** | **1.4** | **6** | **167** | **M** | **56** |
| 7.93 | **4.82** | **307** | **12.85** | **1.8** | **1** | **8.6** | **298** | **M** | **35** |
| 7.93 | **4.82** | **307** | **12.85** | **1.8** | **0.9** | **7** | **198** | **M** | **42** |
| 7 | **5.57** | **285** | **10.9** | **5.3** | **3** | **8.2** | **650** | **M** | **50** |
| 8.4 | **3.88** | **337** | **9.3** | **5.5** | **2.6** | **5.9** | **384** | **M** | **89** |
| 10.3 | **4.6** | **324** | **10.3** | **12.9** | **1.21** | **8.2** | **2.19** | **M** | **55** |
| 15.9 | **3.9** | **519** | **10.3** | **2.6** | **1** | **5.3** | **180** | **M** | **60** |
| 4.8 | **3.4** | **340** | **15.8** | **1.1** | **0.8** | **6.9** | **145** | **F** | **55** |

***Table S1: The data table contains medical and hematological data for 42 patients.***

2) ***Supplementary Analysis: Subgroup Stratification and Disease Severity***

The dataset contains 43 rows in total (Attached in the supplementary file ***Table S1: The data table contains medical and hematological data for 42 patients.***).

It appears to be a clinical dataset likely used to study the relationship between systemic inflammation (NLR), immune system responses (WBC differentials), and metabolic conditions (like diabetes indicated by HbA1c/Glucose) across different age groups and sexes.

Based on the dataset compiled by the authors, the data table contains medical and hematological data for 42 patients. It focuses on complete blood count (CBC), kidney function tests, diabetes markers, and basic demographic information. The following is a breakdown of the specific data grouped into fifteen columns:

1) Complete Blood Count (CBC) and Differential Analysis:
- RBC: Red blood cell count.
- WBC: White blood cell count.
- Platelet count.
- Hb: Hemoglobin level.
- NLR: Neutrophil-to-lymphocyte ratio (a commonly used indicator of systemic inflammation).
- E, B, M, L, N: These represent the percentages of differential white blood cell counts, corresponding to eosinophils, basophils, monocytes, lymphocytes, and neutrophils.
  2) Metabolic and Functional Indicators:
- Creatinine: An indicator used to assess kidney function.
- Glycated hemoglobin (HbA1c): An important indicator of long-term blood glucose control (used to diagnose or monitor diabetes).
- Glucose: Blood glucose level.
  3) Demographics:
- Sex: Patient's biological sex (male/female)**.**
